# Stereotactic radiofrequency ventral posterolateral thalamotomy for cancer pain

**DOI:** 10.3171/2020.7.FOCVID2023

**Published:** 2020-10-01

**Authors:** Abteen Mostofi, Ali Rezaei Haddad, Fotios Bourlogiannis, Erlick A. C. Pereira

**Affiliations:** 1 Neurosciences Research Centre, St. George’s, University of London; and; 2 St. George’s University Hospital, London, United Kingdom

**Keywords:** cancer pain, stereotactic, lesion, thalamus, radiofrequency

## Abstract

Palliative neuroablative procedures are often performed for medication-refractory cancer pain. A 57-year-old female with lung carcinoma and metastases to the brachial plexus and cervical spine with severe neuropathic pain affecting the right upper limb was referred to the authors’ functional neurosurgery service. This video shows her treatment with an awake stereotactic radiofrequency thalamotomy targeting the left ventral posterolateral nucleus. Postoperatively, she experienced immediate and complete resolution of the pain. Palliative radiofrequency thalamotomy can be a viable and effective procedure for somatotopically distributed regional cancer pain.

The video can be found here: https://youtu.be/jykYWXTP3c4

## Transcript

**0:29 Introduction.** We present the case of a 57-year-old female with a right apical lung cancer with metastases to the brachial plexus and cervical spine. She was referred to our functional neurosurgery service with medication-refractory severe right upper-limb neuropathic pain which preceded her cancer diagnosis by several months. She had received chemo-, radio-, and immunotherapy and had an estimated survival of 18 months at the time of referral. The pain was stabbing in nature and affected the entire limb, radiating from the shoulder to the hand. It was associated with dysesthesia, allodynia, and hyperalgesia. Her right hand was nonfunctioning and placed in a splint. In this video, we show the patient undergoing a stereotactic radiofrequency lesion of the ventral posterolateral thalamus,^1,2^ which was successful in improving her pain. The choice of thalamotomy over other neurosurgical options was governed by the need for a minimally invasive palliative procedure with rapid onset of effect, and also by the well-localized but regional nature of the pain, which rendered the sensory thalamus an attractive target to achieve coverage of the affected limb. Awake surgery not only helps intraoperative assessment to target the affected body region but avoids risks of general anesthesia in a patient with advanced lung cancer.

**1:48 Preoperative Preparation.** A Cosman-Roberts-Wells stereotactic frame is applied under local anesthesia.

The CT localizer is secured to the frame base ring.

The plane of the base ring approximates the infraorbitomeatal plane.

A stereotactic CT scan is performed, avoiding the base ring and incorporating all of the localizer bars. The scan is checked for quality before the patient is extracted from the scanner table.

**2:27 Stereotactic Planning.** The target is defined on a preoperative planning MRI scan obtained a few weeks prior to surgery. The anterior and posterior commissures are identified.

**2:39** The ventral posterolateral thalamic target is marked 13 mm lateral to the posterior commissure in the intercommissural plane, or equidistant from the midline and medial border of the posterior limb of the internal capsule, contralateral to the side affected by pain.^3^ Somatotopically, the upper-limb area of the ventral posterior thalamus lies between the face area medially and the lower-limb area laterally.^4^

**3:08** A secondary target in the centromedian-parafascicular complex is marked in case of failure of the primary target;^5^ this is 5 mm anterior and 2 mm medial to the ventral posterolateral thalamic target.

**3:25** The stereotactic CT scan is uploaded to the planning software and fused with the planning MRI scan.

After confirmation of satisfactory image fusion, the stereotactic frame fiducials are detected automatically by the planning software and the target coordinates are expressed in stereotactic space.

**3:47 Positioning.** The patient enters the operating room and lies supine, with the frame fixed to the operating table.

**3:54 Stereotactic Arc Setup.** The stereotactic arc is assembled and set to the desired target coordinates, ring, and arc angles.

The accuracy of the trajectory is confirmed using a phantom target.

**4:10 Surgical Procedure.** The patient’s hair is washed with chlorhexidine-based skin cleanser and prepared with alcoholic chlorhexidine solution. The stereotactic arc is mounted on the frame base ring. The operative field is draped.

A probe dipped in methylene blue dye is used to mark the skin incision site.

Minimal hair removal around the incision site is achieved with a scalpel.

Local anesthetic is infiltrated and a 2-cm linear skin incision is made.

A methylene blue–tipped stereotactic probe is again used to mark the skull entry point, and a handheld drill used to make a small pilot hole in the outer table.

A handheld 2.7-mm-diameter twist drill mounted on the stereotactic arc is used to make a small craniostomy.

A 1.8-mm-diameter radiofrequency probe with an exposed 2-mm tip is introduced through the craniostomy to the primary ventral posterolateral thalamic target.

The probe is connected to a radiofrequency generator.

**5:40 Intraoperative Assessment.** A stepwise exploration is performed from 2 mm above to 2 mm below the target and in parallel tracks 2–4 mm medial and lateral if required depending on somatotopy. At each step, test stimulation is applied at 100 Hz and pulse width 0.5 ms and the patient asked to report the presence and distribution of induced sensations such as paraesthesia or analgesia. Stimulation at 2 Hz and pulse width 0.5 ms is also used to assess for capsular motor side effects. Stimulation amplitude is increased in steps of 0.5 V. The site with lowest amplitude threshold for sensory coverage of the painful body part with highest threshold for side effects is selected to lesion. Lack of sensory coverage at 2 V or presence of side effects at 1 V are generally indications to explore adjacent targets.

If stimulation at and adjacent to the ventral posterolateral thalamus is ineffective or has too low a threshold for side effects, then a lesion there is not performed and the secondary centromedian-parafascicular locus is targeted with stimulation and target exploration before consideration of a lesion.

At an intensity of 1 V the patient reports paraesthesia affecting the right upper limb, spreading to the trunk and face, and covering the painful area.

A thermal radiofrequency lesion is made. The probe tip is heated to 80°C for 60 seconds.

The probe is removed and the incision closed.

**7:40** Postoperatively, the patient was immediately aware of an improvement in the right upper-limb pain but experienced a transient dysarthria lasting less than 24 hours. MRI of the brain on the first postoperative day confirmed the location of the left thalamic lesion. She was discharged on the third postoperative day completely free of pain with unchanged analgesic medication. She died 2 months after the procedure.

The ventral posterolateral thalamic nucleus is an effective target in deep brain stimulation for noncancer pain of regional somatotopic distribution, such as neuropathic pain from brachial plexus avulsion or amputation.^3^ A lesion of this nucleus is likewise a palliative option for cancer pain with a similar body part distribution. Cordotomy is an alternative procedure for such regional pain, while cingulotomy is suited to more diffuse whole or hemibody pain.^6^

## Author Contributions

Primary surgeon: Pereira. Assistant surgeon: Bourlogiannis. Editing and drafting the video and abstract: Mostofi, Rezaei Haddad, Pereira. Critically revising the work: Rezaei Haddad, Bourlogiannis, Pereira. Reviewed submitted version of the work: Rezaei Haddad, Bourlogiannis, Pereira. Approved the final version of the work on behalf of all authors: Mostofi. Supervision: Mostofi, Pereira.
